# The Effectiveness of an Online Nutrition Education Program on Varsity Athletes’ Nutritional & Dietary Supplement Knowledge

**DOI:** 10.3390/nu17010044

**Published:** 2024-12-27

**Authors:** Jana Daher, Margo Mountjoy, Dalia El Khoury

**Affiliations:** 1 Department of Family Relations and Applied Nutrition, University of Guelph, Guelph, ON N1G 2W1, Canada; jdaher@uoguelph.ca; 2 Department of Family Medicine, McMaster University, Hamilton, ON L8P 1H6, Canada; mountjm@mcmaster.ca

**Keywords:** nutrition education, dietary supplements, varsity athletes, sports nutrition, education intervention

## Abstract

**Background/Objectives**: Research has shown that athletes often have poor nutritional knowledge, particularly regarding dietary supplements. The purpose of this study was to investigate the effectiveness of an online nutrition education program in improving nutritional and dietary supplement knowledge among varsity athletes at the University of Guelph in Ontario, Canada. **Methods**: A total of 30 varsity athletes at the University of Guelph were randomized into experimental [n = 18] and control [n = 12] groups. A randomized wait-list controlled intervention was used, where participants in the experimental group were granted access to an online, 4-week nutrition education program, while no program was provided to the control group within the duration of the study. The program covered nutrition topics and focused on sports nutrition and dietary supplementation. Both groups were administered a validated online nutrition and dietary supplement questionnaire at baseline and post-intervention. **Results**: Mean baseline knowledge scores for the experimental and control groups were 13.78 ± 2.76 and 13.92 ± 2.39, respectively, and were not significantly different [*p* = 0.888]. Post-intervention scores were 16.28 ± 1.49 and 14.5 ± 1.88 for the experimental and control groups, respectively, [*p* < 0.05]. There was a significant interaction between the intervention and time on knowledge. **Conclusions**: These results indicate that this nutrition education program was successful in significantly improving nutritional and dietary supplement knowledge in varsity athletes.

## 1. Introduction

Nutritional knowledge is a key factor impacting food choices and modifying dietary habits [1]. Adequate nutrition and informed dietary practices are critical for athletes to achieve optimal performance. Despite training in environments where resources and information sources needed for optimal performance are usually available, research has shown that athletes still follow suboptimal diets and have poor nutritional knowledge, especially regarding dietary supplementation, and lack awareness of recommendations [1,2,3]. Several studies have reported that athletes rely on unreliable sources of information with regard to supplementation [4], such as family and friends [5], teammates, coaches, the internet, or their own judgment [6]. While healthcare professionals were the top source of information for athletes in some studies [7], other studies have shown that physicians were ranked eighth while dietitians were ranked sixteenth [8].

A large body of evidence has shown that nutrition education interventions can improve nutritional and dietary supplement knowledge and address gaps in both athletes [1,9,10] and non-athletes [11,12,13]. Interventions differed in terms of their delivery format, duration, frequency, and curriculum. A systematic review by Tam et al. (2019) found that nutrition education interventions were mainly delivered in an in-person format, were short-term (typically lasting less than 4 weeks), and varied in session frequency (most often weekly) and duration (usually 1 h or less) [1]. Only a few studies used an online format. Since this review was published before the COVID-19 pandemic, it is likely that the number of studies examining the effectiveness of online interventions has increased, although no studies are currently available to confirm this. While face-to-face interventions were proven to be effective in improving nutritional knowledge, there are several limitations, such as scheduling challenges, logical constraints, access barriers, and higher expenses. Additionally, these interventions often require significant time and resources from both participants and facilitators, which can limit scalability and consistency in delivery. A systematic review by Murimi et al. (2019) investigated factors that contribute to effective online nutrition education interventions, which can address the limitations of in-person approaches [14]. The study identified six factors: (1) the use of tailored messages and/or individualized feedback; (2) participant engagement; (3) intervention duration ≥ 3 months; (4) identification of specific targeted behaviors; (5) alignment of intervention activities with stated objectives; (6) and use of theory-based interventions [14]. Despite their convenience, online nutrition education interventions have limitations such as inadequate engagement of participants, potential bias due to relying on self-reported data, and failure to maintain long-term intervention outcomes [14].

While many nutrition education interventions focused on general nutrition knowledge, only a few of them focused on sports nutrition and dietary supplements knowledge in athletes [9,10]. This topic is particularly important in light of the lax guidelines controlling dietary supplement production and distribution. Since athletes are more likely to use sports supplements, it is vital that they understand any associated risks, mainly supplement contamination and inadvertent doping [15,16].

Therefore, this study implemented an online nutrition education program developed for varsity athletes at the University of Guelph and aimed to investigate its effectiveness in terms of improving their nutritional and dietary supplement knowledge.

## 2. Materials and Methods

### 2.1. Study Design

**Figure 1:**
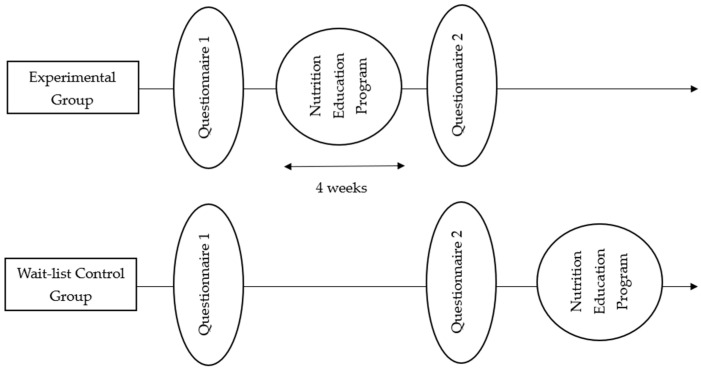
Experimental procedure.

A randomized wait-list controlled intervention design was used. A wait-list control group serves as an untreated comparison group during the study, but eventually goes on to receive the same intervention. The wait-list control group was used in order not to deny participants in the control group access to the intervention, while providing an untreated comparison for the experimental group. In this study, the intervention group participants were granted access to an online, 4-week nutrition education program, while no program was provided to the control group within the duration of the study. The control group participants, for ethical purposes, were granted access to the program after they filled out the second questionnaire (Figure 1). An electronic informed consent was obtained from all participants prior to participation. This study was approved by the University of Guelph Research Ethics Board (REB# 22-03-003).

### 2.2. Participants

Participants were athletes from different varsity teams at the University of Guelph. Posters were placed in high-traffic and social areas at the University of Guelph’s campus, particularly in the Athletic Center and the Health and Wellness Center. Emails were sent to team captains, coaches, and strength and conditioning staff inviting athletes to participate in this study. Posters were also shared on the varsity teams’ Instagram accounts. After participants were assessed for eligibility (part-time or full-time university students, members of a varsity team, and fluent in English), they were randomized into the intervention or the wait-list control group through an online randomization tool. A total of 30 athletes were recruited (18 in the intervention group; 12 in the control group). The sample size was based on previous similar studies that recruited a similar number [3,17]. Participants were informed that participation is voluntary, and that all data collected would be kept strictly confidential. After completing the study, participants received a $50 gift card as a token of appreciation.

### 2.3. Nutrition Education Program

**Figure 2:**
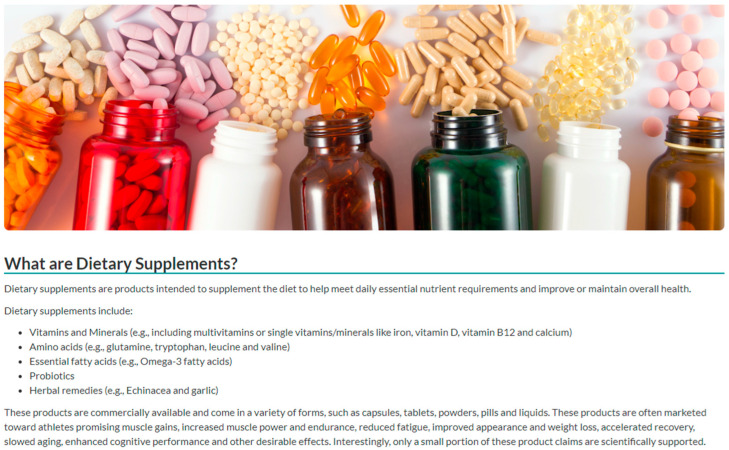
A snapshot from the “dietary supplements” unit on Courselink.

The nutrition program was developed by the research team and was published on Courselink, the University of Guelph’s learning management system. The course, titled “ Nutrition for Athletes: A Focus on Dietary Supplements” consisted of 4 units, where each unit covered a topic. Units were written in lay language, and included quizzes, myth busters, and fun facts (Figure 2). Each unit’s reading time was around 15–20 min.

The topics of the units in the interventional program were (1) Nutrition in Sports, (2) Water and Hydration in Sports, (3) Dietary Supplements, and (4) Risks Associated with Dietary Supplement Use. Each unit consisted of an introduction and learning outcomes page, main information section, summary, quiz, and unit resources. The learning outcomes for each unit are outlined in Table 1. The first unit, “Nutrition in Sports”, covered information on energy, carbohydrates, protein, fat, and timing nutrition before, during, and after exercise. The second unit on water and hydration covered details about dehydration, ways to identify if one is dehydrated, and methods for preventing dehydration. The third unit on dietary supplements covered the definition of dietary supplements, examples, and their uses, along with their categorization. The final unit, entitled “Risks Associated with Dietary Supplement Use”, focused on topics such as doping and fair play in sports, inadvertent doping, safety, quality, and the marketing of dietary supplements. It also discussed the risks associated with the use of dietary supplements and provided suggestions on how to use them safely. Participants were encouraged to complete one unit per week; however, it was up to the participants to complete them at their own pace. Before sending the post-program questionnaire to the experimental group participants, their progress in Courselink was monitored. Email reminders were sent to participants who did not complete the 4 units before sending them the post-program questionnaires.

### 2.4. Questionnaire

All participating athletes filled out an online questionnaire twice. Athletes assigned to the intervention group filled out the questionnaire before and after completing the nutrition education program, which was administered over 4 weeks. Athletes in the control group filled out the questionnaire twice, 4 weeks apart.

The questionnaire used in this study was adapted from a previous study and has been tested for validity and reliability on the target population. Content validity was examined by receiving feedback from a panel of subject matter experts, who evaluated the questionnaire for 12 factors, including brevity, length, number of items, clarity, repetition, order, precision of language, response options, scale, bias, double-barreled questions, and appropriateness of questions [18]. The post-intervention questionnaire included an additional section on suggestions to improve the program.

The “demographics” section included questions on the participant’s age, gender, medical condition, level of education of parents or guardians, ethnic background, smoking habits, and alcohol consumption, while the “assessment of knowledge” section included 18 questions: 6 true/false and 12 multiple-choice questions on dietary supplements, sports nutrition, hydration, and health. For instance, one of the questions about dietary supplements was “True or False: Dietary supplements should be taken by all athletes to improve athletic performance and optimize their health”. Another question related to sports nutrition was, “When should protein be ingested to enhance and accelerate glycogen repletion”? The options given were pre-physical activity, during physical activity, early post-physical activity, and later post-physical activity.

### 2.5. Statistical Analysis

The values of the measured variables will be presented as mean and standard deviation (±SD), unless otherwise stated. Descriptive statistical analysis was used to determine the mean, standard deviation, frequency, and percentage. A two-way mixed analysis of variance (ANOVA) was conducted using the statistical software package for social sciences SPSS (v.24 for MS Windows) after checking for assumptions. Outliers were assessed by examination of studentized residuals for values greater than ±3. The normality of residuals was checked through Normal Q-Q Plots. Homogeneity of variances was assessed by Levene’s test of equality of error variances, and homogeneity of covariances was assessed by Box’s test of equality of covariance matrices. For the analysis, a 95% confidence interval was used (*p* ≤ *0*.05).

## 3. Results

### 3.1. Participants’ Characteristics

**Figure 3:**
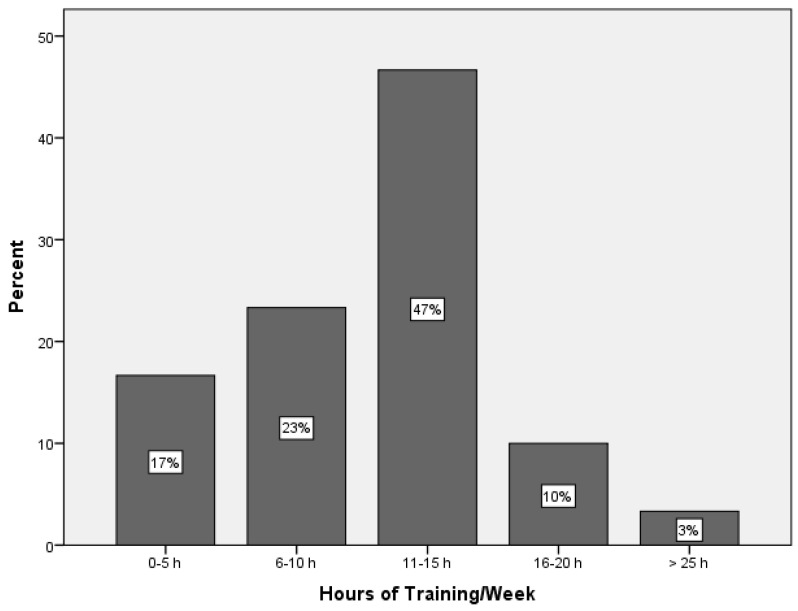
Weekly training hours of participants.

The age of participants ranged from 18 to 30 years old, with a mean age of 20.6 ± 2.9. The majority identified as female, while males accounted for 30% of the sample. The majority of participants identified as Caucasian, followed by Southeast Asian and West Asian. None of the participants reported that they were currently smoking. Around half of the participants reported that they were currently consuming alcohol. The most frequently reported level of parents’/guardians’ education was a bachelor’s degree (43.3%) (Table 2). Participants were also asked about their training time per week. In total, 47% trained for 11–15 h per week, while only 3% trained for more than 25 h per week (Figure 3).

### 3.2. Knowledge

There were no outliers, as assessed by studentized residuals for values greater than ±3. The data were normally distributed, as assessed by Normal Q-Q Plots. There was homogeneity of variances (*p* > 0.05) and covariances (*p* > 0.001), as assessed by Levene’s test of homogeneity of variances and Box’s M test, respectively. Means and SD for knowledge scores are presented in Table 3.

**Figure 4:**
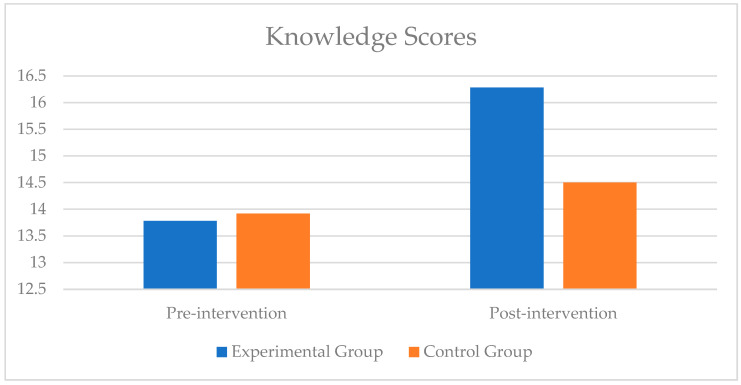
Change in knowledge scores at baseline (pre-intervention) and post-intervention for control and experimental arms.

There was a statistically significant interaction between the intervention and time on knowledge, F(1, 28) = 4.47, *p* = 0.043, and partial η^2^ = 0.138 (Table 4). Simple main effects for group analyses revealed that there was no statistically significant difference in knowledge scores between the experimental and the control group at baseline, but knowledge scores were statistically significantly greater in the experimental group (M = 16.28, SE = 0.39, *p* = 0.007) compared to the control group after the intervention (Figure 4).

Simple main effects for time revealed a statistically significant effect of time on knowledge scores for the experimental group, F(1, 17) = 14.66, *p* = 0.001, and partial η^2^ = 0.463, as knowledge scores, were statistically significantly higher at post-intervention (M = 16.28, SE = 0.35, *p* = 0.001) compared to pre-intervention (M = 13.78, SE = 0.65, *p* = 0.001). There was no statistically significant effect of time on knowledge scores for the control group.

## 4. Discussion

The aim of this study was to examine the effect of an online nutrition education program on varsity athletes’ knowledge about dietary supplements and sports nutrition. Knowledge scores of athletes in both groups were similar at baseline. Participants in the experimental group showed a significant increase in knowledge scores from 13.78 ± 2.76 at baseline to 16.28 ± 1.49 after the intervention. There was no significant change in knowledge scores for the control group. These results indicate that the interventional nutrition education program was successful in improving nutritional and dietary supplement knowledge in varsity athletes. The absence of baseline group differences further attributes the improvement in knowledge scores to the intervention, which is not unexpected. A systematic review on the effectiveness of education interventions designed to improve nutritional knowledge in athletes found that most education interventions reported a significant improvement in nutritional knowledge, despite variations in delivery format, duration, frequency, contact time, and curriculum [1]. A 7-week education intervention consisting of lectures, group discussions, and group activities improved sports nutrition knowledge among team sports athletes [9]. A study by Rossi et al. found that attending a 90 min nutrition session improved sports nutritional knowledge in NCAA Division I baseball players [10].

Furthermore, this positive impact is not limited to athletes. Several other studies investigating the effects of nutrition education interventions on different target populations have also reported significant improvements in knowledge. For example, interventions with adolescents [13], college students [11], teachers, learners [12], and high school students from low-income communities [3] all demonstrated similar positive outcomes.

The majority of nutrition education interventions used face-to-face education [1]. This delivery format may be less accessible for individuals with time constraints or those who are unable to attend in person, which could limit the reach and impact of the intervention [14]. This method can also be time-consuming for both athletes and educators, impacting its practicality and sustainability [10]. With the rise in internet use worldwide and the enhanced accessibility and time flexibility of online education, online nutrition education programs are expected to become more widely used. There was no attrition in this study, which likely exists in technology-based interventions [1]. This intervention’s success can also be attributed to being self-paced, written in lay language, including interactive elements, and, most importantly, published through the University of Guelph’s learning management system, which students are already familiar with. Moreover, participants’ engagement was tracked, and the second questionnaire was only administered after all four units were completed.

Some studies have questioned whether nutrition education interventions can sustain outcomes over time. While our study did not include a follow-up questionnaire, similar studies have reported long-term knowledge retention and behavior sustainability at one-month [19], three-month [20], four-month [21,22], and six- to eight-month follow-ups [23].

Several limitations of this study should be considered when interpreting the results or planning future research. Relying on self-reported data may introduce potential bias. Since this was an online intervention, participants were unable to ask questions or seek clarification, which may have limited their understanding of the material. Knowledge retention was not investigated due to the lack of a follow-up component. Additionally, future research could benefit from a larger sample size and from a more diverse sample, since the majority of this study’s participants were Caucasian. Finally, because the majority of participants were females, the study was unable to explore potential sex-based differences.

## 5. Conclusions

In conclusion, the present study demonstrates that “Nutrition for Athletes: A Focus on Dietary Supplements”, an online nutrition education program designed for varsity athletes, was successful in improving nutritional and dietary supplement knowledge in varsity athletes. These results support the need for and the effectiveness of nutrition education interventions in improving athletes’ nutritional knowledge, which may also enhance their dietary behaviors. This program may serve as a framework for future studies, especially in athletic settings, and would support the development and implementation of similar programs.

Further research is needed to explore the long-term effects of similar interventions in terms of knowledge retention as well as the impact on dietary practices and supplement use over time.

## Figures and Tables

**Table 1:** Learning outcomes of the Nutrition for Athletes program.

Unit Number	Learning Outcomes
Unit 1	Describe the impact and importance of adequate nutrition in sports. Explain what factors influence nutritional intake relating to exercise. Time your nutritional intake to enhance athletic performance and prevent discomfort. Explain the importance of adequately fueling your body.
Unit 2	Describe the importance of adequate hydration in sports. Identify the signs of dehydration. Implement adequate hydration practices before, during, and post-exercise. Explain factors that influence fluid needs.
Unit 3	Identify the purpose of dietary supplements. Describe the effects certain dietary supplements have on athletic performance. Recognize that dietary supplement consumption is not always necessary.
Unit 4	Describe the risks associated with dietary supplement use. Make informed decisions about current and future dietary supplement use. Critically evaluate the claims made by dietary supplement products. Use WADA, and other academic resources to find information about dietary supplements.

**Table 2:** Socio-demographic characteristics of participants.

Characteristics	Prevalence
*Participant Age (n = 30)*	
Mean ± SD	20.6 ± 2.9
Range	18–30
*Gender*	
Male	30%
Female	70%
*Ethnicity*	
Caucasian	66.7%
Southeast Asian	10%
West Asian	10%
Indigenous	3.4%
Latin	3.3%
Arab	3.3%
Prefer not to disclose	3.3%
*Smoking*	
Yes	0%
No	100%
*Alcohol Consumption*	
Yes	53.4%
No	43.3%
Prefer not to disclose	3.3%
*Parents’/Guardians’ Level of Education*	
University diploma above bachelor’s degree	36.7%
Bachelor’s degree	43.3%
College certificate or diploma	16.7%
High school diploma or equivalent	3.3%

**Table 3:** Knowledge scores’ means and SD for control and experimental arms.

Knowledge Scores *	Pre-Intervention	Post-Intervention
Experimental group	13.78 (2.76)	16.28 (1.49)
Control group	13.92 (2.39)	14.5 (1.88)

* Maximum knowledge score is 18.

**Table 4:** Summary of 2 × 2 mixed ANOVA results for knowledge scores (those marked with an asterisk were significant at the 0.05 probability level).

		Simple Main Effects			
Outcome Measure	Time × Group Interaction	Time in Control Group	Time in Experimental Group	Group at Time 1	Group at Time 2
Knowledge	F(1, 28) = 4.47, *p* = 0.043, partial η^2^ = 0.138 *	F(1, 11) = 1.286, *p* = 0.281, partial η^2^ = 0.105	F(1, 17) = 14.66, *p* = 0.001, partial η^2^ = 0.463 *	F(1, 28) = 0.02, *p* = 0.888, partial η^2^ = 0.001	F(1, 28) = 8.32, *p* < 0.05, partial η^2^ = 0.229 *

## Data Availability

The original contributions presented in the study are included in the article. The questionnaire is available upon request from the corresponding author.
